# Vertical compressive bearing performance and optimization design method of large-diameter manually-excavated rock-socketed cast-in-place piles

**DOI:** 10.1038/s41598-023-41483-w

**Published:** 2023-08-30

**Authors:** Xiangmei Zhao, Nan Yan, Xiaoyu Bai, Songkui Sang, Xiaoyu Chen, Yamei Zhang, Mingyi Zhang

**Affiliations:** 1 School of Civil Engineering, Qingdao University of Technology, 777 Jialingjiang Road, Qingdao, 266520 Shandong China; 2 The Hong Kong polytechnic university the department of civil and environmental engineering, Kowloon, 999077 China

**Keywords:** Civil engineering, Petrology

## Abstract

To study the vertical compressive bearing characteristics of large-diameter rock-socketed cast-in-place piles, eight manually-excavated rock-socketed cast-in-place piles were subjected to vertical compressive on-site load and pile stress tests. The test results showed that the load–displacement (*Q-s*) curves of the eight test piles were all slow-varying, and the settlement of the piles was less than 11 mm, which met the minimum engineering requirements. The unloading rebound rate was between 55 and 75%, and the elastic working properties of the piles were apparent. The pile axial force gradually decreased with depth, and the slope of the axial force distribution curve reached a minimum in the moderately weathered muddy siltstone layer while the pile side friction resistance reached its maximum value. Pile end friction increases with the increase of load. But the pile end resistance was inversely proportional to the single pile length-to-diameter (L/D) ratio and the depth of rock embedment for the pile. The percentage of pile side friction resistance under maximum load was 86%, indicating that these were characteristic friction piles. Based on the test results and the current Chinese code, the friction coefficient of the pile side soil layer *η* and the total resistance coefficient of the rock-socketed section *ζ* were introduced. A revision to the calculation equation for the vertical bearing capacity of the rock-socketed cast-in-place pile in the code was proposed, together with an optimization design method for large-diameter rock-socketed cast-in-place piles.

## Introduction

As a particular pile type embedded in the rock foundation, the rock-socketed cast-in-place piles have a high bearing capacity, low cost, low environmental pollution and public hazards, and better adaptability to the geological strata than other pile types. They are widely used in large projects such as bridges, wharves, and high-rise buildings^1–4^. However, the complexity of rock foundations has become a challenge for engineering design^5^. Therefore, it is critical to study the bearing characteristics of large-diameter rock-socketed cast-in-place piles under vertical loads for engineering practice.

Several model tests and numerical and theoretical analysis methods have been proposed in the engineering community for investigating the ultimate bearing capacity of rock-socketed piles under vertical loads. Dai et al.^6^ conducted load tests on ten rock-socketed model piles to study the load transfer response of the piles under different pile-rock interface roughness conditions. Eid et al.^7^ and Ai et al.^8^ and Singh et al.^9^ and Kim et al.^10^ and Maniam et al.^11^ investigated the settlement characteristics of vertically loaded piles and the bearing deformation characteristics of rock-embedded piles using the finite element method (FEM). Seol et al.^12^ established a numerical model for rock-embedded bored piles considering the load transfer effect. From their results, they obtained the pile-soil contact surface's slip and shear load transfer characteristics. Jeong et al.^13^ proposed a shear load transfer function for piles. They presented an analytical algorithm to study the load transfer characteristics of rock-embedded bored piles under axial load. However, these analyses have overlooked several essential aspects: (1) It is difficult for model tests to simulate the actual distribution of soil and rock layers and the complexity of pile-soil-rock interactions. (2) Geotechnics is characterized by diversity and complexity. Prediction methods are based on idealized models and must be verified for reliability compared to field tests. (3) There are too many assumptions in the theoretical calculations, which makes the theoretical analyses have a particular gap with the soil's actual force and deformation characteristics.

Although there is currently a relatively complete understanding of the development of the bearing capacity of rock-socketed piles, this is insufficient for designers to continue using non-field specific empirical correlations to estimate socketed rock pile bearing capacity values^14^. These issues can be addressed by high-quality static load tests, which are considered to be the most reliable and fundamental method for determining the vertical ultimate bearing capacity of rock-socketed piles^15,16^. Akgüner and Kirkit^17^ performed static load tests on seven rock-socketed monopiles and compared the vertical bearing capacity obtained from the tests with the estimated vertical bearing capacity from empirical methods. It was found that the vertical compressive load capacity obtained by empirical methods was conservative compared with the pile load tests. Basarkar and Dewaikar^18^ and Kulkarni and Dewaikar^19^ analyzed the load transfer characteristics of rock-embedded piles under axial loads based on field-measured data. Furthermore, some field tests have shown that optimizing the design of rock-socketed rock piles can significantly save construction and labor costs^20^. For example, in recent years, more than 200,000 rock-socketed piles have been used to construct high-speed railways, highways, large-span bridges, and other infrastructure in Guizhou Province, China. If the length of each rock-socketed pile could be reduced by 1 m, more than 3 million USD in construction costs could be saved^21^.

Design standards usually adopt different calculation methods for bearing capacity, so the coefficient of pile resistance of any two standards may differ substantially^22–26^. The current Chinese code, “Technical code for building pile foundations” (JGJ94-2008)^27^, has a simple and straightforward method for calculating the vertical ultimate bearing capacity of rock-socketed piles. It can directly reflect the influence of the uniaxial compressive strength of rock and rock-socketed depth on the bearing capacity of the rock-socketed section. However, four aspects should be improved in this code: (1) The overall coefficient value in the specification is the comprehensive value of end resistance and side resistance. The end resistance and side resistance coefficients of the rock-socketed section are determined separately, which differs from the true character of the rock-socketed section side resistance and end resistance to jointly exert vertical bearing capacity; (2) There is no precise classification of bedrock types, which may result in errors in the value of comprehensive coefficients; (3) The code values are conservative; (4) Considering only the stress of rock-socketed pile in the ultimate state is inconsistent with the service performance of pile foundation in the working state in practical engineering.

In response to the above issues, this paper illustrated the vertical load transfer law of large-diameter manually-excavated rock-socketed cast-in-place piles through on-site static compressive load tests. Under the condition of satisfying the bearing characteristics of the pile foundation, the scientific and feasibility of the design could be significantly improved with the help of optimal design of the pile foundation to achieve the purpose of cost reduction and efficient construction. Two factors were introduced: the friction coefficient of the pile side soil layer *η* and the total resistance coefficient of the rock-socketed section *ζ*. The bearing capacity calculation formula in the Chinese code was modified so that the calculation formula was closer to the actual working conditions.

## Engineering background

**Figure 1:**
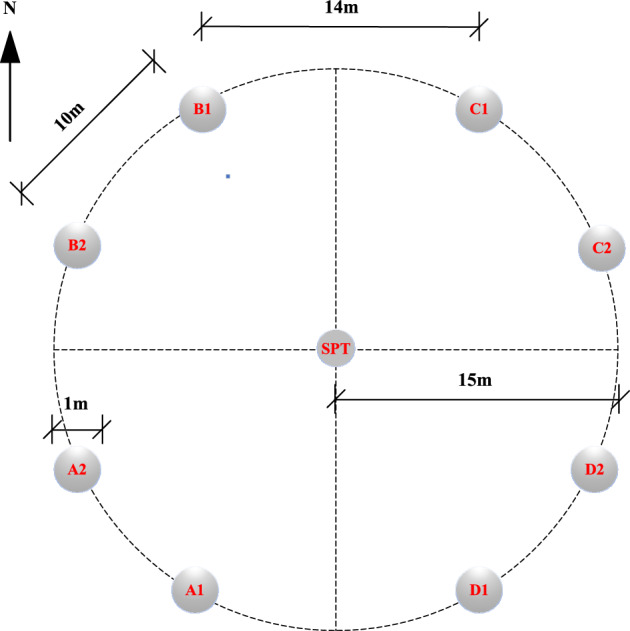
Location of the test piles and SPT on site.

**Figure 2:**
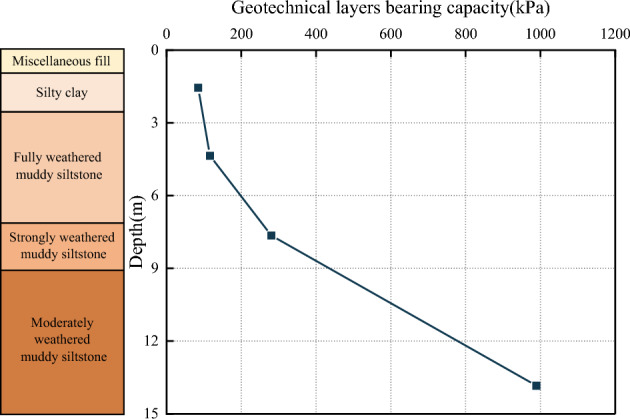
Bearing capacity of geotechnical layers.

**Figure 3:**
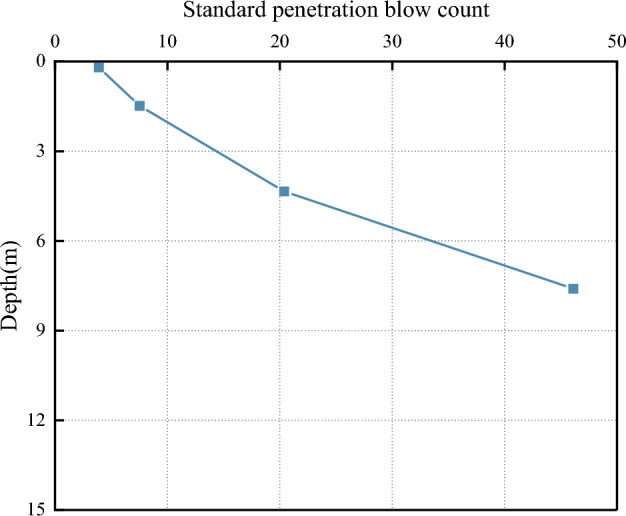
Standard penetration hammer blows.

The test is based on a project in Qingdao; the site’s topography is gentle overall. The site is a denuded and deposited quasi-plain landscape, which has since been modified by artificial backfilling. The stratigraphic structure of the site is simple, and the sequence of layers is clear. The distribution of geotechnical layers from top to bottom is miscellaneous fill, silty clay, fully weathered muddy siltstone, strongly weathered muddy siltstone, and moderately weathered muddy siltstone. The bedrock is mainly a muddy siltstone block of the Wang’s Group Red Earth Cliff Formation of the Cretaceous group, with sandy debris accounting for about 80% of the total rock. The rock’s main mineral composition is potassium feldspar (30%), plagioclase (20%), quartz (20%), and black mica (10%), containing mud debris, heavy minerals, and other mineral fragments, with some of the feldspars undergoing sericitization. As a result of long-term internal and external geological stresses, weathering zones with varying engineering properties have been formed from top to bottom. The maximum groundwater level at the test site was approximately 9.0 m below natural ground level at the time of testing. On-site tests such as Standard Penetration Tests (SPT), flat plate load tests, and rock point load tests were conducted to obtain indices of the geotechnical layers’ bearing capacity, standard penetration blow count, and uniaxial compressive strength of the rock. Laboratory tests on in-situ and sand samples obtained the physical and mechanical parameters such as water content, natural unit weight, pore ratio, liquid-to-plastic limit, and compression modulus. The location of the test piles (denoted as TP) and the SPT test on site are shown in Fig. 1. The physical and mechanical parameters of each stratum at the building site are shown in Table 1, Figs. 2 and 3.

**Table 1:** Physical and mechanical indices of the geotechnical layers at the test site.

Layers	Thickness (m)	*f*_*rk*_ (MPa)	*E* (MPa)	*γ* (kN·m^–3^)	*c*_*k*_ (kPa)	*φ*_*k*_ (°)	SPT N-values
Cultivated soil	0.3–2.1	–	–	18.0	–	15.0	4
Miscellaneous fill	0.4–4.5	–	–	18.0	–	18.0	8
Silty clay	0.3–2.5	–	6.80/*E*_S_	19.2	32.4	13.5	21
Fully weathered muddy siltstone	0.6–4.8	–	7.32/*E*_S_	19.4	38.2	16.2	46
Strongly weathered muddy siltstone	1.5–9.5	3.5	20.0/*E*_0_	22.0	–	35.0*	–
Moderately weathered muddy siltstone	9.5–35	5.2	40.0/*E*_0_	24.0	1012	39.3*	–

*f*_*rk*_ is the uniaxial compressive strength of the rock obtained from the rock point load test (MPa); *E* is the modulus of elasticity of the soil (MPa), *E*_*S*_ is the modulus of compression of the soil (MPa), *E*_*0*_ is the modulus of deformation of the soil (MPa), *γ* is the natural unit weight, those values were obtained by laboratory tests on in-situ and sand samples; *c*_*k*_ is the cohesion, *φ*_*k*_ is the angle of internal friction, the *c*_*k*_ and *φ*_*k*_ values were obtained by an unconsolidated and undrained triaxial test.

*is the equivalent angle of internal friction.

As can be seen from Table 1, Figs. 1 and 2, the values of the physical and mechanical parameters of each stratum, foundation bearing capacity and standard penetration blow count of each geotechnical layer gradually increase with increasing depth of burial and reach their maximum values in the moderately weathered muddy siltstone rock layer. The test results show that with the increase of soil depth, the soil bearing capacity and stability of the test site strata were increased. The rock layer demonstrating relatively strong stability and the highest bearing capacity was the moderately weathered muddy siltstone. Therefore, the moderately weathered section of the rock layer would be the most suitable pile foundation-bearing layer.

## Test plan and process

### Overview of pile configurations

**Figure 4:**
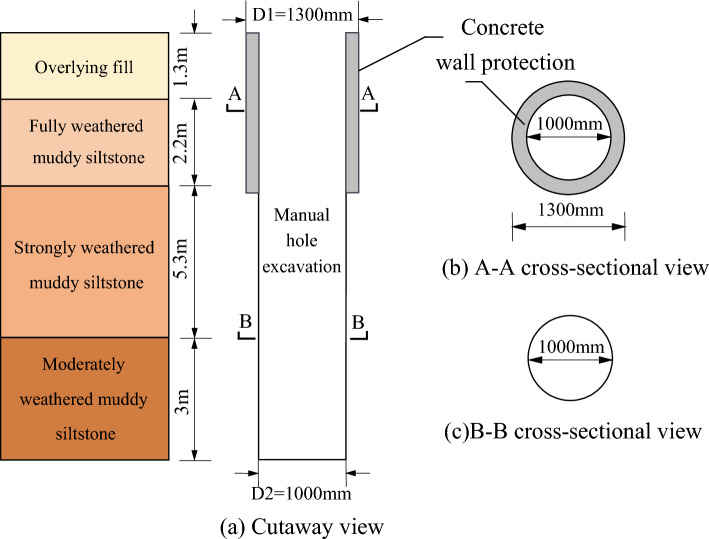
Diagram of test pile dimensions.

This project requires piled foundations and has selected large-diameter manually-excavated rock-socketed cast-in-place piles. The construction process of the piles consisted of the manual excavation of circular holes through the soil layers and rock until the required design depth was attained. The side walls of the holes were protected from collapse by installing a 150 mm thick concrete wall, which was constructed using a supporting mold for every 1 m of excavation. The concrete wall protection was used in the overlying fill and fully weathered muddy siltstone layers. The pile reinforcement cage was placed in the hole, and the hole was filled with C30 strength grade concrete. The eight test piles were distributed in four sites, A, B, C, and D (refer to Fig. 1), in response to the varying terrain. Two test piles were arranged in each area, with diameters of 1.0 m and pile lengths varying between 9 and 14 m. The primary reinforcement consisted of 12 Grade 3 steel bars with diameters of 16 mm. Spiral stirrups with diameters of 12 mm and spacing of 100 mm were used for the first 1.5 m from the top of the pile, while for the remainder, spiral stirrups with a diameter of 12 mm and spacing of 200 mm were used. Ring-welded stiffening stirrups of 14 mm diameter and 2000 mm spacing were arranged along the entire length of the test pile. The dimensions of the test piles are shown in Fig. 4.

**Figure 5:**
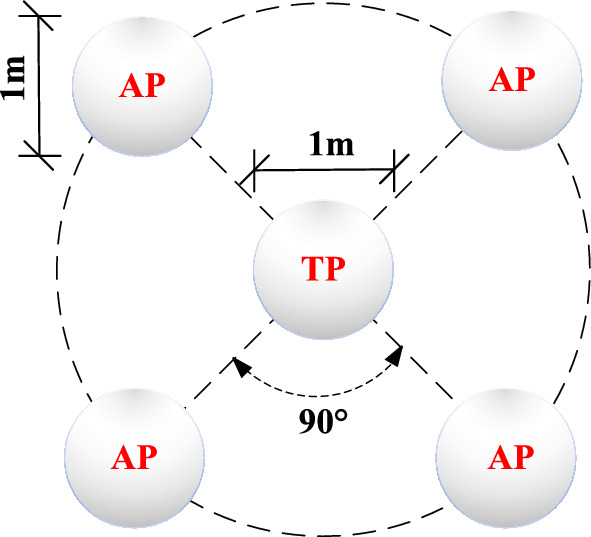
Distribution of test pile (TP) and anchor pile (AP) field areas.

The anchor pile method was used for the static load test. The anchor piles were symmetrically placed around the test piles in a cruciform manner. The anchor piles were manually-excavated enlarged base piles with a diameter of 1.0 m and an expanded bottom diameter of 2.0 m. The depth of embedment in the weathered bedrock was 1.3 m. According to current Chinese code, “Technical code for building pile foundations” (JGJ94-2008)^27^, the piles’ vertical pull-out ultimate bearing capacity was 4050 kN, and the concrete strength grade was C30. The test and anchor pile site distribution and test pile design parameters are shown in Fig. 5 and Table 2, respectively.

**Table 2:** Test pile parameters.

Test pile number	Pile length (m)	Overlying fill thickness (m)	Fully weathered muddy siltstone thickness (m)	Strongly weathered muddy siltstone thickness (m)	Moderately weathered muddy siltstone thickness (m)
A1	11.8	1.3	2.2	5.3	3.0
A2	9.8	0.9	2.4	3.5	3.0
B1	11.5	2.0	3.5	2.7	3.3
B2	12.1	2.5	3.0	4.0	2.5
C1	12.5	2.6	3.6	3.1	3.3
C2	12.8	2.9	3.4	3.5	3.0
D1	12.5	2.3	2.8	4.2	3.2
D2	13.5	2.6	2.8	4.0	4.1

### Sensor installation and arrangement

**Figure 6:**
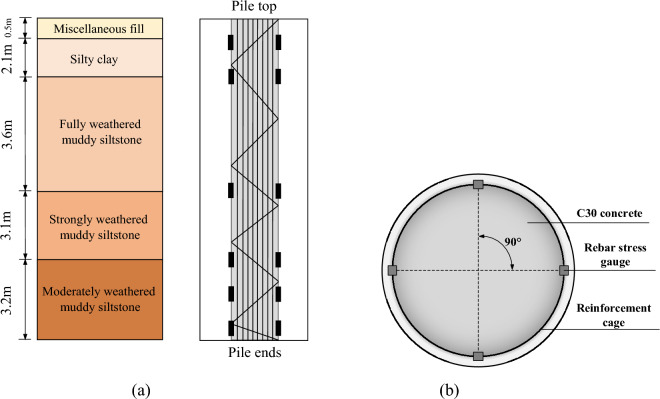
Schematic diagram of the reinforcement stress gauge distribution: (**a**) Cutaway view. (**b**) Cross-sectional view.

**Figure 7:**
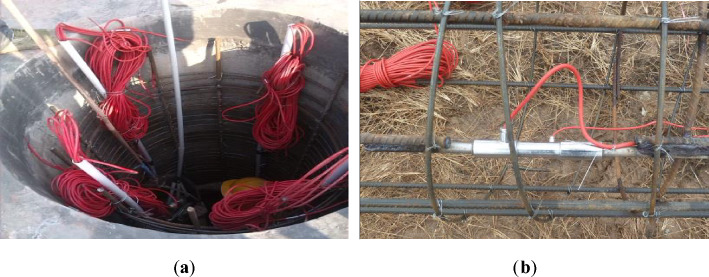
Site photograph of the sensor arrangement: (**a**) Pile top steel stress gauge wire layout. (**b**) Coaxial lap welding photo.

To improve the accuracy and operability of the test, JTM-V1000 vibratory rebar stress gauges were installed in corresponding locations on the body and on top of each of the test piles. Six sets of rebar stress gauges were installed on each test pile, each set consisting of four symmetrically installed stress gauges on each section, as shown in Fig. 6. The gauge sets were positioned at the soil boundary layers, starting at 0.5 m from the top of the pile and at each boundary layer until the moderately weathered muddy siltstone. Thereafter, one set was positioned halfway between the moderately weathered muddy siltstone boundary and the base of the pile, and one at the base. Therefore, each test pile was fitted with 24 rebar stress gauges, with a total of 192 stress gauges being installed. The rebar stress gauges were coaxially lap welded to the main reinforcing steel cage before placement in the excavated pile holes. The frequency values of the rebar stress gauges were recorded before and after each loading stage to measure any changes in pile axial force, side friction resistance, and end resistance resulting from the loading. The arrangement of the rebar stress gauges and the installation of the field sensors are shown in Figs. 6 and 7.

### Low-strain test

Before the pile settlement and stress tests, the test piles and anchor piles were tested for pile integrity using the low-strain method. The low-strain on-site test used a vibration hammer (type DFC-2) to excite the pile at the top center position to transmit stress waves downward along the pile body. The acceleration speed sensor (type RS-V1411) had a sensitivity of 233.6 mv/(cm/s) and received the vibration signals during excitation, while a pile foundation dynamic testing instrument amplified the stress waves transmitted to the pile top. The dynamic testing instrument (INV-306UDLF-3) had an amplification factor of 1–10,000, a frequency response of 0–20 kHz, and a minimum sampling interval of 10 ns.

Based on the current Chinese code “Technical Code for Testing of Building Foundation Piles” (JGJ 106-2014)^28^, the integrity quality grades of the test piles were assessed; the results are shown in Table 3. From the low-strain test results, the test piles and anchor piles were all Class I piles.

**Table 3:** Low-strain test results.

Test pile number	Length (m)	Test wave speed (m/s)	Integrity type
A1	11.8	3710	I
A2	9.8	3640	I
B1	11.5	3650	I
B2	12.1	3690	I
C1	12.5	3670	I
C2	12.8	3650	I
D1	12.5	3750	I
D2	13.5	3470	I

Tested single piles with intact pile bodies are classified as Class I piles, those with minor defects as Class II piles, those with apparent defects as Class III piles, and those with severe defects as Class IV piles.

### Static load test device

**Figure 8:**
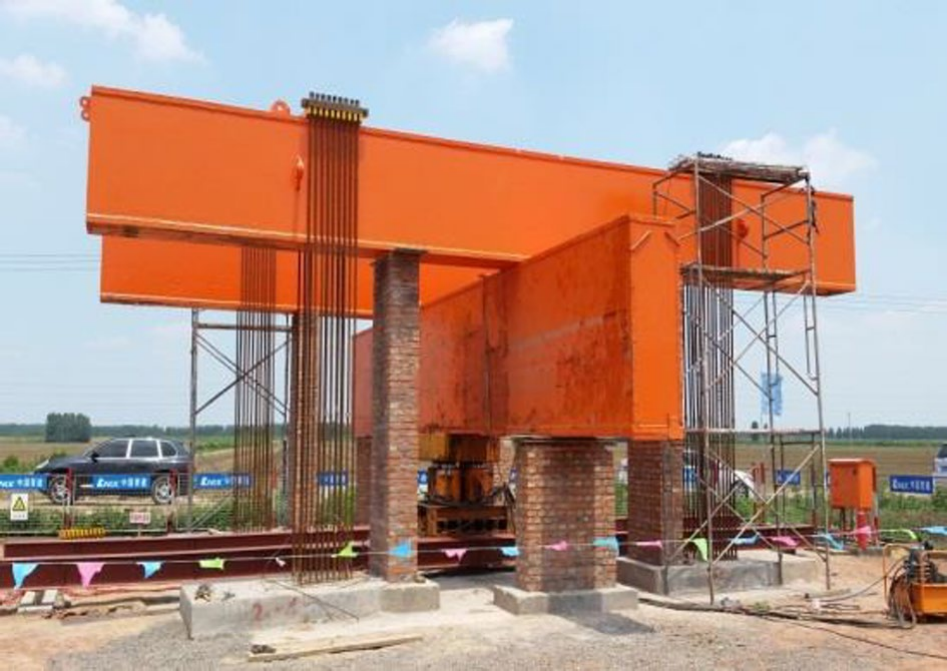
Photograph of the pile static load test equipment.

The single pile static load compressive test consisted of reaction force, loading, load measuring, and displacement measuring devices. The reaction force was provided by the anchor piles, and box-shaped steel main and secondary beams. Two symmetrically arranged 5000 kN hydraulic jacks were used for graded loading. The ST3000 pressure sensor (with a range of 3000 kN and an error of less than 0.1% of full scale) was used to measure the applied load for each loading stage at the pile top. The RSWS-50 series displacement sensor measured the top displacement during the loading process, with a displacement sensor range of 50 mm. Four settlement observation points were fixed at the top of each test pile and guided to a stable reference point using a reference beam. Using the JTM-V1000 vibratory rebar stress gauge and JTM-V10B frequency reading instrument (with an accuracy of less than 0.05% FS and ± 0.1 Hz, respectively), the stress of each pile section was measured and recorded during the graded loading process. A site photograph of the test pile static load test is shown in Fig. 8.

## Static load test results

### Load-settlement (Q-s) curve

In general, the settlement at the top of a pile directly reflects the pile's load-bearing performance and transfer mechanism. Therefore, in engineering research, the ultimate bearing state of piles is mainly determined based on the top settlement^29^. According to the Chinese code^27^, the maximum loading of the static load test should be greater than twice the characteristic value of the pile's compressive bearing capacity. From the Eq. (1) of the characteristic value of vertical compressive bearing capacity of monopile, the characteristic value of the test piles A1, A2, B2, C1 and C2 was 6480 kN, and that of the test piles B1, D1, D2 was 5400 kN. The characteristic value is defined as the bearing capacity value obtained by dividing the maximum load resisted by a single pile before failure by a safety factor of 2.0, while the maximum loading is the bearing capacity of a single pile before instability or significant deformation occurs. The maximum loading for test piles A1, A2, B2, C1 and C2 was 12,960 kN, and 10,800 kN for test piles B1, D1 and D2. In accordance with the provisions of the “Technical Code for Testing of Building Foundation Piles” (JGJ 106-2014)^28^, this test adopted a graded and equal loading approach, with an initial loading value of 2160 kN for all the test piles. Due to the large maximum loading capacity of test piles A1, A2, B2, C1 and C2, the loading increase for each stage was 1080 kN, up to a maximum load of 12,960 kN. The loading increase for each stage for test piles B1, D1, and D2 was 2160 kN, up to a maximum load of 10,800 kN. None of the eight test piles showed significant damage when loaded to these maximum loads.

1 $$ {\text{F}} = {\text{Q}}_{{\text{t}}} \times B \times {\text{L,}} $$

where, *F* is the characteristic value of vertical compressive capacity of a single pile, *Q*_*t*_ is the compressive strength at the top of the pile, *B* is the diameter or length of the pile, and *L* is the height of the bottom of the pile foundation.

**Figure 9:**
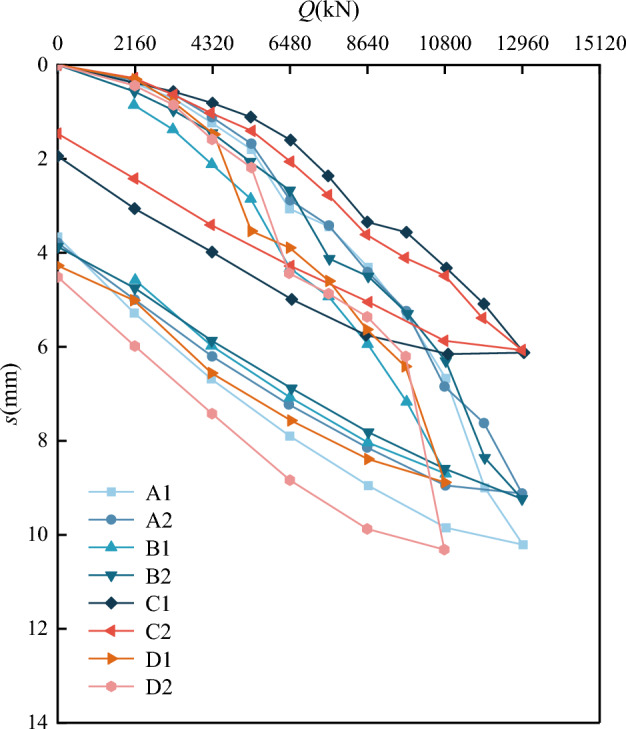
*Q-s* curves of the test piles.

Figure 9 shows the *Q-s* curves of the eight test piles, while Table 4 indicates each test pile’s static load test results and settlement under the maximum loads. From Fig. 9, it can be seen that the settlement at the top of the eight test piles ranged from 6 to 11 mm. These settlement values were less than the maximum of 40 mm allowed by the Chinese code^27^, indicating that the pile foundations embedded in soft rock could meet the engineering design and use requirements and thus have specific bearing potential. In addition, these results verify the feasibility of the manual excavation pile process under such geological conditions. As indicated in Table 4, under the maximum load, the top settlement of test pile C2 was the smallest (6.07 mm). However, the rebound after unloading accounted for a significant proportion of the settlement, such that pile C2 had a rebound rate of 75%, reducing the residual settlement to 1.50 mm. In contrast, the top settlement of test pile D2 was the largest but with a smaller unloading rebound rate (55%), indicating that the plastic deformation of test pile D2 was more significant than the plastic deformation of test pile C2.

**Table 4:** Static load test results.

Test pile number	Maximum loading value (kN)	Pile settlement (mm)	Rebound settlement (mm)	Residual settlement (mm)	Rebound rate (%)
A1	12,960	10.17	6.44	3.73	63.3
A2	12,960	9.11	5.28	3.83	58.0
B1	10,800	8.67	5.52	3.15	63.7
B2	12,960	9.20	5.32	3.88	57.8
C1	12,960	6.21	4.21	2.00	67.8
C2	12,960	6.07	4.57	1.50	75.3
D1	10,800	6.91	4.10	2.81	59.3
D2	10,800	10.31	5.72	4.59	55.5

**Figure 10:**
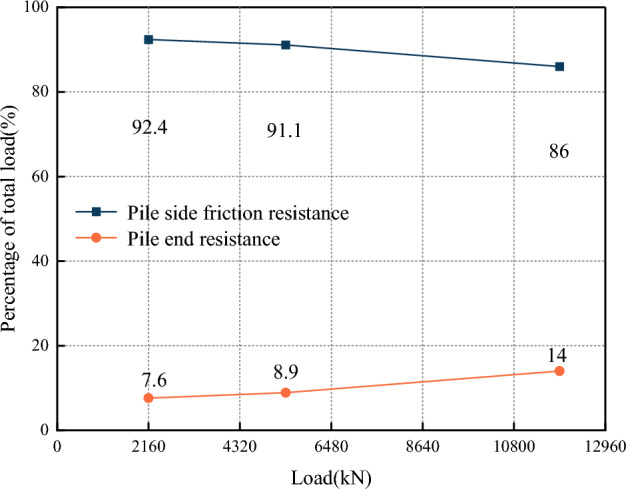
Proportional sharing of applied load between average pile end resistance and side friction resistance.

Under the initial load (2160 kN), working load (5404 kN), and maximum load (10,800 kN and 12,960 kN), the average pile end resistance and pile side resistance of the eight test piles shared the proportion of the applied load, as shown in Fig. 10.

The resistance to the initial load (2160 kN) was shared between the side friction resistance and end resistance by 92.4% and 7.6%, respectively, indicating that the load was predominantly carried by the side friction resistance. As the applied load was increased to its maximum, the side friction resistance’s portion dropped to 86%, and the end resistance's portion rose to 14%. At maximum load, the load was predominantly carried by the side friction resistance for all the test piles. Therefore, all eight test piles exhibited the characteristics of friction piles^27^, which is consistent with the research results of Dai et al.^30^ and Kou et al.^31^. The reason for the relatively large proportion of side friction resistance was analyzed: the effective pile length of the test pile was longer, which increased the pile-rock contact area, and then increased the side friction resistance. The pile end resistance was difficult to be utilized, which made the side friction resistance bear a more significant proportion of the pile top load.

### Pile axial force distribution pattern

The force value of a single steel bar under loads can be calculated using the following equation:

2 $$ {\text{F}}_{ij} = K\left( {f_{0}^{2} - f_{i}^{2} } \right), $$

where *K* is a sensor constant, *f*_0_ is the initial frequency before loading, *f*_*i*_ is the output frequency resulting from loading.

Assuming that the relative displacement of concrete and reinforcement under the applied load was zero during the test, indicating that the strains were the same, this can be written as:

3 $$ \varepsilon_{s} = \varepsilon_{c} , $$

where *ε*_*s*_ is the strains of the reinforcement, and *ε*_*c*_ is the strain of the concrete.

The axial forces at the different pile sections under the varying applied loads can be obtained from the stress–strain relationship as follows:

4 $$ {\text{N}}_{ij} = \frac{{\left| {F_{ij} } \right| * E_{i} * A_{p} }}{{E_{s} * A_{s} }}, $$

5 $$ E_{i} = \frac{{Q * E_{s} * A_{s} }}{{A_{s} * F_{i1} }}. $$

In these equations, *N*_*ij*_ is the axial force of the *j* layer section of the test pile at the *i* loading stage (kN), *F*_*ij*_ is the load value of the reinforcement obtained from the sensor frequency at the cross-section of layer *j* under the *i* loading stage (kN), *E*_*i*_ is the equivalent pile body elastic modulus under the *i* loading stage (kN/m^2^), *A*_*p*_ is the cross-sectional area of the pile body (m^2^), and *A*_*s*_ is the cross-sectional area of the rebar steel (m^2^). In Eq. (4), *F*_*i*1_ is the load value of the steel reinforcement obtained from the sensor frequency at the first layer cross-section under the *i* loading stage (kN), and *Q* is the applied load (kN).

**Figure 11:**
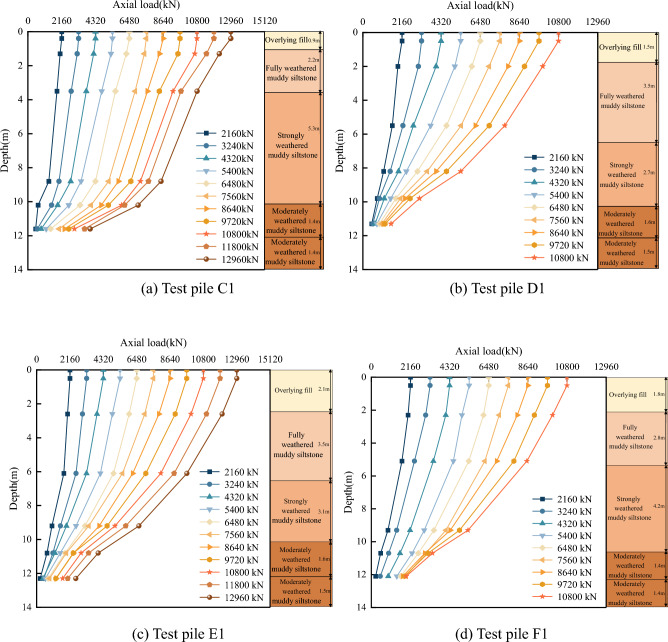
Distribution of test pile axial force with depth.

Using the above axial force calculation method, the calculation results were plotted as a pile axial force distribution curve along the length of the pile. For illustrative purposes, only the distribution curves for four test piles, A1, B1, C1, and D1, are shown in Fig. 11.

As shown in Fig. 11, the axial force distribution curves of the four test piles are similar, indicating no apparent difference between the geological conditions and foundation bearing capacity of the four sites. In addition, the similarity verifies the feasibility of this test scheme and the reliability of the data. As previously noted, the pile side friction resistance carried most of the applied load; therefore, for each load stage, the axial force distribution decreased with increasing pile depth, ending close to zero at the end of the pile. As the load increased, the slope of the distribution curve decreased, with the largest decrease occurring in the moderately weathered muddy siltstone layer. This corroborates the findings that the side friction resistance of each pile-soil and pile-rock layer played a more significant role as the load increased.

### Pile side friction distribution pattern

Based on in-situ static load tests, and assuming that the pile side friction resistance is uniformly distributed between two adjacent sensors in the longitudinal pile direction, the side friction resistance and end resistance under various loading stages can be calculated using Eqs. (6) and (7).

6 $$ q_{i} = \left( {N_{i} - N_{i - 1} } \right)/\left( {U_{i} U_{p} } \right), $$

7 $$ q_{b} = \frac{{Q - Q_{s} - Q_{r} }}{{\pi D^{{^{2} }} }}, $$

where *q*_*i*_ is the pile side friction resistance of the *i*-th layer (kPa), *N*_*i*_ and *N*_*i*-1_ are the pile axial forces above and below the *i*-th layer (kN), *U*_*i*_ is the thickness of the *i*-th layer (m), and *U*_*P*_ is the test pile circumference (m). In Eq. (6), *q*_*b*_ is the pile end resistance (kPa), *Q*_*s*_ is the total pile soil resistance (kN), *Q*_*r*_ is the total pile rock resistance (kN), and *D* is the pile body diameter (mm).

**Figure 12:**
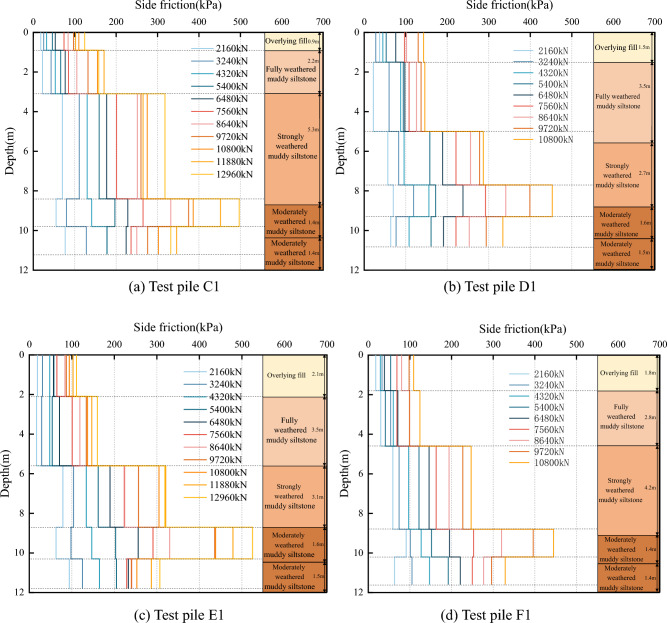
Distribution curve of side frictional resistance with pile depth.

**Figure 13:**
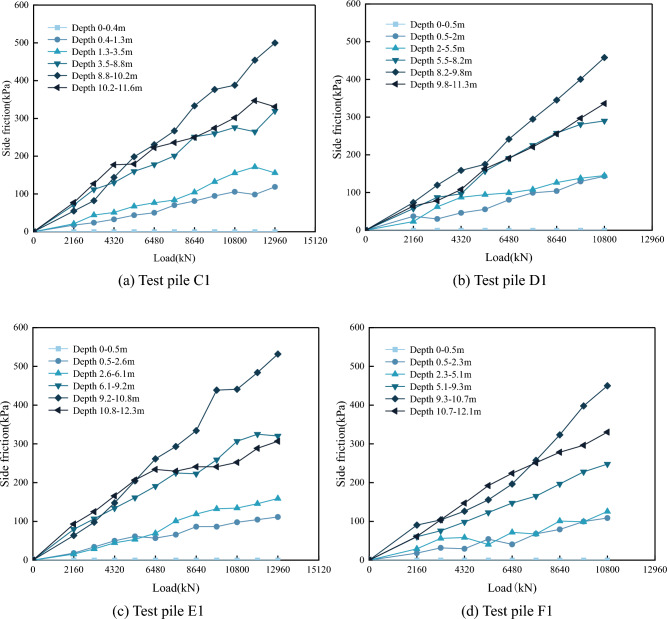
Distribution curve of pile side frictional resistance with applied load.

The results of the calculations were plotted as distribution curves of the pile side friction resistance with depth and load stages. For illustrative purposes, only the results for test piles A1, B1, C1 and D1, are presented here, as shown in Figs. 12 and 13.

As shown in Fig. 12, the side friction resistances show top-down distributions, where the effective contributions are influenced by the nature and burial depth of the soil layers. Under the initial load, the peak values were not apparent, although the side friction resistance values were more prominent in the strongly weathered muddy siltstone and moderately weathered muddy siltstone layers. As the applied loading increased, each stratum's friction resistance value increased to different degrees. The peak values were more noticeable with increased loading, with the peak values occurring in the upper moderately weathered muddy siltstone layer. At the maximum load, the side resistance was 450 ~ 500 kPa. In contrast, the side resistance at the pile end was not fully developed, only attaining 280 ~ 340 kPa, indicating differences in the side resistance at smaller stratigraphic depths (3.0 m).

The side resistance values presented in Fig. 13 show varying rates of change with increasing loads. The most significant increase occurred in the rock embedment section, particularly after the third loading stage. The side resistance of the rock embedment section increased from 150 to 550 kPa, indicating an increase greater than 200%; in contrast, the overlying soil layers showed an increase in the side resistance of less than 100%. Under loading, the contributory degree of side resistance at different burial depths varied: for the fully weathered muddy siltstone layer, the pile side resistance was close to the ultimate value at the beginning of loading (approximately 150 kPa); the strongly weathered muddy siltstone layer reached its ultimate value (approximately 200 kPa) at the seventh load stage 7th (7560 kN); and the deeper moderately weathered muddy siltstone layer had no significant turning point in the pile side friction resistance values. This indicates that the pile side resistance of the upper fourth soil layer was fully utilized under maximum load, while the rock embedment section (moderately weathered muddy siltstone) had the potential for further utilization.

### Pile end resistance distribution pattern

**Figure 14:**
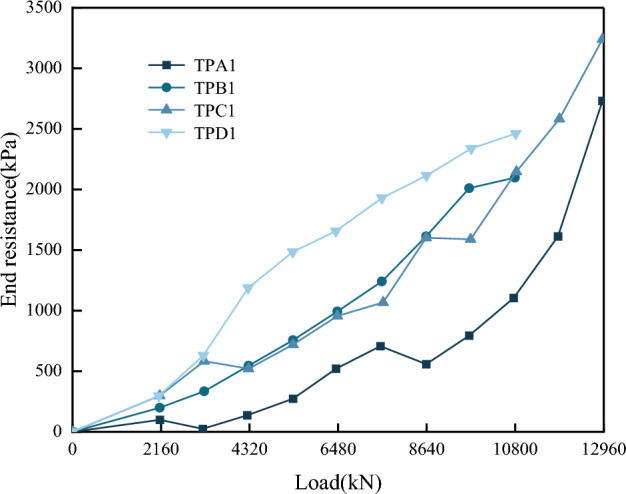
Distribution of pile end resistance with applied load.

Figure 14 shows the variation of pile end resistance with applied load for test piles A1, B1, C1, and D1.

As shown in Fig. 14, pile end resistance increased as the applied load increased, as well as the rate of change, indicating that the end resistance became fully effective as the load increased. When the applied load increased to 6480 kN, the pile end resistance increased almost linearly with the applied load.

**Figure 15:**
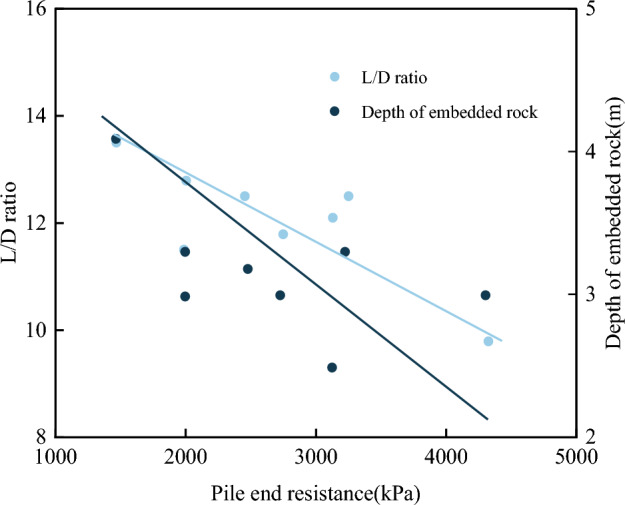
End resistance versus test pile L/D ratio and rock embedment depth.

In this instance of eight test piles with the same diameter, the pile end resistance was inversely proportional to the single pile length-to-diameter (L/D) ratio and the depth of rock embedment for the pile, as shown in Fig. 15. The decrease in pile end resistance with the increase of effective pile length and rock-embedment depth is consistent with the findings of Li et al.^32^, who studied the bearing characteristics of short piles embedded in a weathered rock base in Qingdao from field static load tests. Increasing the L/D ratio and increasing the depth of rock embedment showed the same mechanism. Due to the increase in effective pile length or rock-embedment depth, the contact area between the piles and the surrounding rock increased, thereby increasing the side friction resistance. In contrast, the proportion of pile end resistance carrying the applied load decreased, preventing the pile end resistance from being used to its full potential. Thus, it can be deduced that increasing the pile length or rock-embedment depth will increase construction difficulty and the project cost without utilizing the full bearing capacity potential of the pile end resistance. Therefore, the design parameters of piled foundations should be optimized for each project.

## Optimized design methods

### Comparison of field results with design code recommendations

**Figure 16:**
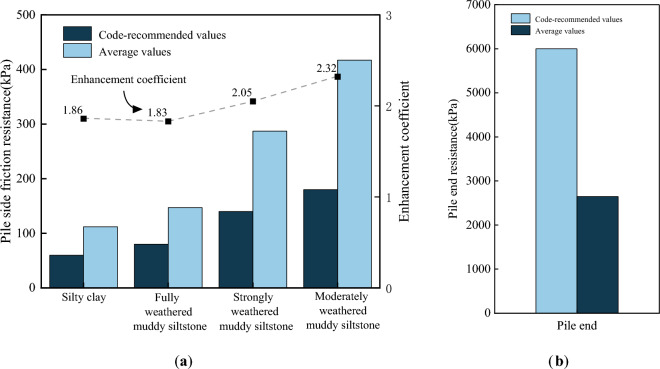
Comparison of test pile values versus code-recommended values: (**a**) Average side friction resistance; (**b**) Average pile end resistance.

Based on the data obtained from the field tests, the average values of pile side friction resistance and end resistance at the maximum load were compared with the design code recommended values, as shown in Fig. 16. The design recommended values were determined from the current Chinese code^27^ based on the site conditions. The measured data is realistic, reliable, relevant, and has reference significance.

As already mentioned, as the depth of pile burial increased, the measured values of side friction resistance of the eight test piles increased. As Fig. 16a indicates, these average values varied significantly with the varied depths. The overlying soil layer (silty clay) had the lowest side resistance, while in contrast, the moderately weathered muddy siltstone layer had the highest side resistance. Thus, the resistance of the moderately weathered muddy siltstone layer was approximately four times the side resistance of the overlying fill, three times the side resistance of the fully weathered muddy siltstone, and one-and-a-half times the side resistance of the strongly weathered muddy siltstone.

The correlation between the measured and recommended values is defined in this paper as the ratio of the average values of measured pile side friction and pile end resistance to their respective code-recommended values and is termed the enhancement coefficient. The enhancement coefficients increased continuously with the increase in layer depth. The side friction enhancement coefficient of the moderately weathered muddy siltstone was the largest as the measured side friction resistance increased with burial depth (refer to Fig. 12). When the test pile was not loaded to its ultimate condition, the proportion of applied load carried by the side friction force reduced with increasing embedment depth, thus reducing the full utilization of the deep pile side friction. Furthermore, the code recommendations for varying soil and rock formations were substantially smaller than the measured values of side friction resistance, resulting in conservative parameter selections. The code recommendations are based on engineering experience alone and empirical data, disregarding the impact of the wall conditions of manually excavated piles, thus resulting in conservative values. Manually excavated piles effectively increase the contact area between the pile and soil due to the roughness of the pile-soil interface, thus increasing the pile side friction resistance and the enhancement effect with depth. As the design codes do not take this enhancement effect into account, the recommended values are lower than the actual working conditions.

As can be seen from Fig. 16b, the pile end resistance differs from the pile side friction resistance. The measured average value of pile end resistance is about 0.45 times of the code-recommended value. As the end resistance was inhibited by the low applied loads, resulting in the code-recommended values for pile end resistance being substantially greater than the measured average value. However, the pile end resistance gradually increases as the applied load increases. Therefore, similar to the pile side friction resistance, the measured pile end resistance can exceed the code-recommended values when fully utilized.

### Optimization design of large-diameter rock-socketed piles

Under the maximum applied load, the pile settlement was less than 40 mm, and the measured bearing capacity was less than the ultimate bearing capacity of the pile foundation. Based on this data, it was concluded that the test piles still had significant bearing potential, and the pile design parameters were conservative. For manually excavated piles such as those planned for this project, the pile-soil and pile-rock side friction resistance values can currently only be designed according to experience and code recommendations. As these values are conservative, this could increase the construction difficulty and construction cost due to increased pile sizes. Therefore, an optimized design approach was proposed which utilized the pile bearing capacity while maintaining a certain safety reserve. This approach was based on the code and static load test results and considered two aspects, namely, reduction of the pile length or reduction of the pile diameter, and analyzed the economic feasibility of the optimized scheme.

#### Optimization scheme 1: reduction of pile length

The characteristic value of the vertical bearing capacity of a single pile was assumed to be 5404 kN, and the pile side friction and end resistance values were based on the measured values. From this, the minimum pile length that met the working load state was calculated; the optimized results are shown in Table 5. The calculated reduction in pile length was between 59 and 77%. With the maximum length reduction, the optimized pile length was 10.4 m, resulting in the rock-socketed pile no longer being embedded in the weathered rock. With such shallow bedrock embedment, lateral instability would likely occur, thus reducing the pile's bearing capacity. Therefore, this optimization scheme was not deemed suitable.

**Table 5:** Summary of pile length optimization.

Test pile number	Length (m)	Characteristic value (kN)	Optimized pile length (m)	Pile length reduction (%)
A1	11.8	5404	7.0	59.3
A2	9.8	5404	6.8	69.4
B1	11.5	5404	8.2	71.3
B2	12.1	5404	9.0	74.4
C1	12.5	5404	8.2	65.6
C2	12.8	5404	8.3	64.8
D1	12.5	5404	9.1	72.8
D2	13.5	5404	10.4	77.0

#### Optimization scheme 2: reduction of pile diameter

**Figure 17:**
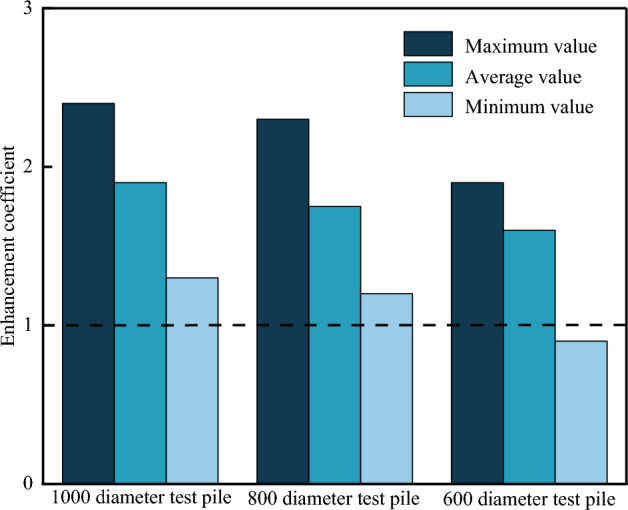
Enhancement coefficient of vertical bearing capacity of piles with varying diameters.

In discussing the effect of pile size on the contribution of pile side friction resistance, Zhang et al.^33^ and Gong et al.^34^ found that when the pile diameter was larger than 800 mm, the pile side friction resistance decreased with increasing pile diameter and increasing rock embedment depth. Therefore, it can be concluded that pile diameters of 800 mm and 600 mm would not affect the development of side friction resistance. Therefore, pile diameters of 800 mm and 600 mm were used in the pile vertical load-bearing limit value calculations. The enhancement coefficient of the vertical bearing capacity of the monopile at different diameters is shown in Fig. 17, where the enhancement coefficient is the ratio of the ultimate bearing capacity of the monopile to the ultimate bearing capacity recommended in the Chinese code^27^.

As can be seen from Fig. 17, when the pile diameter was 800 mm, the ultimate bearing capacity enhancement factor was between 1.5 and 1.8, and the ultimate bearing values met the design requirements with a safety reserve. When the pile diameter was 600 mm, the minimum value of the enhancement factor was less than 1, indicating that this pile diameter would not be suitable for the site conditions. After considering the construction cost and safety factors relative to the test results, the pile diameter of 800 mm was considered the optimal size.

## Calculation of full resistance coefficient

Due to the variability of site geological conditions and construction quality, both the empirical and the theoretical estimation of the bearing capacity of rock-socketed piles could differ significantly from the actual bearing capacities, causing an unnecessary increase in construction costs or safety risks. Therefore, the calculation method for the ultimate bearing capacity of rock-socketed cast-in-place piles should be refined according to the actual working conditions of different sites.

The vertical load-carrying capacity of a single pile with rock embedment rock comprises the total ultimate side resistance of the soil around the pile and the total ultimate resistance of the embedded rock section (the sum of the side friction resistance and end resistance of the moderately weathered muddy siltstone). The equations for calculating the vertical ultimate bearing capacity of rock-socketed piles in the Chinese code, “Technical code for building pile foundations” (JGJ94-2008)^27^, are as follows, those equations are adapted to embedded rock piles with pile ends placed in intact and more intact rock-socketed piles:

8 $$ Q_{uk} = Q_{sk} + Q_{pk} , $$

9 $$ Q_{sk} = U_{p} \sum\nolimits_{i = 1}^{n} {l_{i} } q_{sik} , $$

10 $$ Q_{pk} = \vartheta_{r} f_{rk} A_{p} , $$

where *Q*_*uk*_ is the vertical ultimate bearing capacity of a monopile (kN), *Q*_*sk*_ is the the total ultimate side resistance of the soil around the pile (kN), and *Q*_*pk*_ is the the total ultimate resistance of the embedded rock section (kN). In Eq. (8), *l*_*i*_ is the length of the *i*-th layer of the monopile (m), and *q*_*sik*_ is the ultimate side resistance of the *i*-th layer of the monopile (kN). In Eq. (9), *f*_*rk*_ is the saturated uniaxial compressive strength of the rock, *A*_*p*_ is the area of the pile end (m^2^), and ${\vartheta }_{\mathrm{r}}$ is the comprehensive correction coefficient for the side friction resistance and end resistance of the rock embedment section. The value of ${\vartheta }_{\mathrm{r}}$ is related to the pile-to-rock embedment ratio and the degree of rock softness; the values extracted from the Chinese code^27^ are shown in Table 6. The pile to rock embedment ratio is the ratio of the length of the pile end embedded in the rock to the diameter of the pile body.

**Table 6:** Comprehensive coefficients of side resistance and end resistance of rock-embedded sections.

Pile to rock embedment ratio	0	0.5	1.0	2.0	3.0	4.0	5.0	6.0	7.0	8.0
Extremely soft rock, soft rock	0.60	0.80	0.95	1.18	1.35	1.48	1.57	1.63	1.66	1.70
Harder rock, hard rock	0.45	0.65	0.81	0.90	1.00	1.04				

As the equation given in the Chinese code for calculating the vertical bearing capacity of the rock-socketed pile does not consider the site force conditions of the project, additional factors were introduced. Based on the existing code, the friction coefficient of the pile side soil layer *η* and the total resistance coefficient of the rock-socketed section *ζ* were introduced. Incorporating these coefficients, the equation for calculating the vertical bearing capacity of rock-socketed piles in the existing code was modified to improve the adaptability of the existing code for manually excavated piles. Therefore, the revised equation for the ultimate value of the vertical bearing capacity of rock-socketed piles is as follows:

11 $$ Q_{uk} = \eta Q_{sk} + \zeta Q_{pk} , $$

where the symbols in the equation are the same as previously defined in the text.

Under maximum load, the friction resistance of the soil layer on the pile side and the total resistance of the rock-socketed section were calculated from the measured data from on-site tests. These were compared with the code-recommended values, as shown in Table 7. From Table 7, it can be seen that under maximum load, the average friction coefficient of the pile side soil layer *η* was 1.868, and the average total resistance coefficient of the rock-socketed section *ζ* was 1.303. As the test results showed that the corresponding side friction resistance gradually increased with depth; hence the friction coefficient of the pile-side soil layer *η* was greater than the total resistance coefficient of the rock-socketed section *ζ*. Under the maximum applied load, when the test pile was not at its ultimate limit state, the measured values of the total pile-soil side friction resistance and the total resistance of the rock-socketed section were all greater than the code values. As the measured values were almost twice that of the code values, this indicates that the code values were considerably conservative. Compared with other piling methods, manual hole excavation increases the pile side friction resistance; therefore, the criteria for applying the code values should be clarified. The code value for the total resistance of the rock-socketed section was less than the measured value, indicating that using uniaxial compressive strength of rock as the main parameter of rock formation will lead to conservative results and should thus be amended in the specifications. The above analysis was based on values at the maximum applied load, but the pile side friction resistance and the total resistance of the rock-socketed section will continue to develop as the load increases until the ultimate load is reached. The corresponding friction coefficient of the pile side soil layer *η* and the total resistance coefficient of the rock-socketed section *ζ* will thus also increase further.

**Table 7:** Comparison of specification and measured values.

Test pile number	Maximum load (kN)	Pile settlement (mm)	Pile side soil friction resistance (kN)		Pile side soil friction coefficient *η*	Total resistance of the rock-socketed section (kN)		Total resistance coefficient of the rock-socketed section *ζ*
			Measured values	Specification values		Measured values	Specification values	
A1	12,960	10.17	6908	3367	2.052	6052	5405	1.120
A2	12,960	9.11	4836	2543	1.902	8124	5405	1.503
B1	10,800	8.67	5087	2820	1.804	5713	5561	1.027
B2	12,960	9.20	6952	3350	2.075	6008	5064	1.186
C1	12,960	6.21	6112	3175	1.925	6848	5561	1.232
C2	12,960	6.07	7865	3359	2.341	5095	5405	0.943
D1	10,800	6.91	4985	3324	1.500	5815	5509	1.056
D2	10,800	10.31	4460	3310	1.348	6340	5961	1.064
Average values		8.33	5901	3156	1.868	6249	5484	1.303

**Figure 18:**
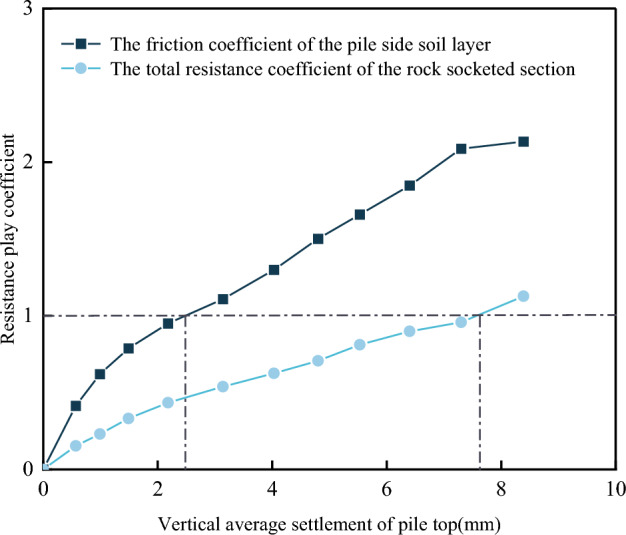
Relationship between the friction coefficient *η* and the total resistance coefficient *ζ* with pile settlement.

To clarify the resistance performance of rock-socketed piles under normal working conditions, the relationship between the vertical pile settlement and the friction coefficient *η* and the total resistance coefficient *ζ* under various loads of 2160 kN, 3240 kN, 4320 kN, 5400 kN, 6480 kN, 7560 kN, 8640 kN, 9720 kN, 10,800 kN, 11,880 kN, 12,960 kN was plotted as shown in Fig. 18.

As seen from Table 7 and Fig. 18, as the settlement of the pile increased, the friction coefficient *η* and the total resistance coefficient *ζ* both increased continuously. During the loading process, the coefficient *η* was always larger than the coefficient *ζ*, and the difference between them gradually increased as the pile settlement increased. The growth rate of the coefficient *η* tended to be higher when the pile settlement was less than 2 mm and slower thereafter. In contrast, the growth rate of the coefficient *ζ* remained relatively constant with no significant variations. When the pile settlement was 2.5 mm, the coefficient *η* had already reached 1.0, indicating that in this state, the measured value of pile-side soil resistance had already reached the recommended value of the design code. Therefore, the pile-side soil resistance had the capacity to increase as the load continued to be applied. In contrast, the total resistance coefficient *ζ* reached a value of 1 when the pile settlement was close to 8 mm. Based on the above analysis, it can be concluded that the size of the friction coefficient *η* and the total resistance coefficient *ζ* can indirectly reflect the proportional size of the applied load shared by the pile side soil friction resistance and the total resistance of the rock-socketed section. At the same time, when the completed building is in use, the limit bearing state (excessive displacement or damage) of the rock-socketed piles is seldom reached in a short period. The practical process of load analysis of the building is thus critical. Therefore, it is recommended that the design of pile foundations consider the performance of pile resistance under different loads, that is, the relationship between the degree of pile resistance performance and applied load or pile settlement during the use of buildings, to further improve the analysis of the vertical compression bearing characteristics of rock-socketed piles.

## Conclusion

Eight large-diameter rock-socketed piles found in muddy siltstone were tested with vertical compressive static loads, based on a critical project in Qingdao. To satisfy the required mechanical and deformation performance, the design feasibility was improved through the optimized design of the pile foundation. The Chinese design code bearing capacity equation was modified by adding two factors, namely the friction coefficient of the pile side soil layer *η* and the total resistance coefficient of the rock-socketed section *ζ*, to render the results closer to the actual working conditions of the project. The main findings of this paper are as follows:

1. The load–displacement (*Q-s*) curves of the eight test piles varied slowly, with pile settlements of less than 11 mm. Under the maximum applied load, the average pile side friction resistance of the eight rock-socketed piles was 86% of the applied load, showing the characteristics of friction piles.
2. Under all loading stages, the pile axial force decreased with increasing depth, and the pile side friction resistance gradually became effective. As the pile load increased, the slope of the pile axial force distribution curve had the flattest slope in the moderately weathered muddy siltstone, while the pile side friction resistance showed the greatest increase. The pile end resistance increased with increasing pile load. For the same diameter, increasing the pile length or the depth of rock embedment of pile is not conducive to the development of pile tip resistance. It provided a warning and engineering design ideas for optimal pile foundation design.
3. The pile side friction resistance contribution varied sequentially from top to bottom, increasing with the applied load and pile depth. The measured value of pile side friction resistance under maximum load was approximately 2.3 times greater than the code-recommended value, indicating that the pile foundation design parameters in the current Chinese code were conservative in these geological conditions. The pile end resistance increased under maximum load, the measured value of pile end resistance was only 0.44 times the code-recommended value, as the pile end resistance did not fully contribute to the load-bearing pile resistance.
4. Based on these test conditions, when the pile foundation design scheme was optimized, it was found that shortening the pile length reduced the rock-socketed pile bearing capacity. In contrast, when the pile diameter was reduced from 1000 to 800 mm, the vertical compressive load capacity of the monopile still had a certain safety reserve.
5. Based on the current Chinese code, the friction coefficient of the pile side soil layer *η* and the total resistance coefficient of the rock-socketed section *ζ* were introduced. Considering the effect of the pile side soil friction and the total resistance of the rock-socketed section, the variation of *η* and *ζ* with pile settlement under different load stages was calculated. For the design of rock-socketed piles, it is recommended that the development of pile side friction resistance and pile end resistance under varying pile loads or settlements be clarified by using the two coefficients, *η* and *ζ*, to improve the understanding of the vertical compressive load bearing characteristics and force mechanism of rock-socketed piles.

## Data Availability

All the data supporting the results are included in the article.
